# Astrocytes in fragile X syndrome

**DOI:** 10.3389/fncel.2023.1322541

**Published:** 2024-01-08

**Authors:** Karo Talvio, Maija L. Castrén

**Affiliations:** Department of Physiology, Faculty of Medicine, University of Helsinki, Helsinki, Finland

**Keywords:** fragile X syndrome, astrocytes, autism spectrum disorder, induced pluripotent stem cells, cholesterol, glutamate, calcium signaling, cell differentiation

## Abstract

Astrocytes have an important role in neuronal maturation and synapse function in the brain. The interplay between astrocytes and neurons is found to be altered in many neurodevelopmental disorders, including fragile X syndrome (FXS) that is the most common inherited cause of intellectual disability and autism spectrum disorder. Transcriptional, functional, and metabolic alterations in *Fmr1* knockout mouse astrocytes, human FXS stem cell-derived astrocytes as well as in *in vivo* models suggest autonomous effects of astrocytes in the neurobiology of FXS. Abnormalities associated with FXS astrocytes include differentiation of central nervous system cell populations, maturation and regulation of synapses, and synaptic glutamate balance. Recently, FXS-specific changes were found more widely in astrocyte functioning, such as regulation of inflammatory pathways and maintenance of lipid homeostasis. Changes of FXS astrocytes impact the brain homeostasis and function both during development and in the adult brain and offer opportunities for novel types of approaches for intervention.

## 1 Introduction

Glial cells comprise major cell populations in the human brain, and astrocytes account for a substantial portion of all glia (von Bartheld et al., 2016). Astroglial cells form functionally, structurally, and developmentally diverse cell populations (Clavreul et al., 2022; Yang et al., 2022). A crucial function of astrocytes is to provide metabolic support to neurons (Turner and Adamson, 2011). They display dynamic activity in the form of intracellular calcium levels, which control the release of neuroactive gliotransmitters (Goenaga et al., 2023). Astrocytes induce the development and destruction of both excitatory and inhibitory synapses and contribute to short- and long-term brain plasticity through distinct mechanisms (Perez-Catalan et al., 2021). Due to their complex arborisation, astrocytes form non-overlapping synaptic islands consisting of an astrocyte and all the synapses it contacts (Halassa et al., 2007). A single astrocyte can influence up to 2 million synapses in the human brain, which facilitates circuit level oversight. Human astrocytes are larger and structurally more complex, and synaptic islands in the human cortex are much larger than those of model animals (Oberheim et al., 2009). Gap junctions allow astrocytes to form networks, whose function is vital for cognition (Hösli et al., 2022). As astrocytes form the blood brain barrier (BBB), they regulate brain water balance through aquaporins (Satoh et al., 2007). By these means astrocytes are in an advanced position to interpret peripheral signals (Lee et al., 2022).

Astrocytes are implicated in various brain pathologies (Lee et al., 2022), including neurodevelopmental conditions such as fragile X syndrome (FXS, Fernández-Blanco and Dierssen, 2020). FXS, first described as an X chromosome-linked syndrome (Martin and Bell, 1943) is considered the most common cause of inherited intellectual disability. Prevalence of FXS is generally quoted as 1 in 4,000 males and 1 in every 6,000–8,000 females. Based on genetic screening studies, the prevalence was estimated to be approximately 1 in every 2,500 regardless of gender (Hagerman, 2008). The FXS symptomatology significantly overlaps with other neuropsychiatric conditions. Approximately a third of FXS patients fulfill diagnostic criteria for autism spectrum disorder (ASD, Harris et al., 2008), and 54–59% meet diagnostic criteria for attention deficit hyperactivity disorder (ADHD, Sullivan et al., 2006). Up to 44% have epilepsy (Cowley et al., 2016), which ranges from generalized seizures to benign focal epilepsy in childhood with centrotemporal spikes (Lozano et al., 2016). In addition, the FXS phenotype includes near-universal stereotypical physical features such as long and narrow face, prominent ears, high-arched palate, prominent jaw, and macroorchidism (Ciaccio et al., 2017).

A CGG trinucleotide repeat expansion in the 5′ untranslated region of the *fragile X messenger ribonucleoprotein 1* (*FMR1*) gene is the most common genetic cause behind FXS (Fu et al., 1991; Verkerk et al., 1991). Varying lengths of the CGG triplet repeat exist. Repeats less than 55 are in the benign range, whereas over 200 repeats lead to embryonic methylation of the promoter, subsequent silencing of the gene, and therefore lack of the *FMR1* protein (FMRP, Sutcliffe et al., 1992). Thus, there exist premutations between 55 and 200 repeats, whose carriers do not exhibit the FXS phenotype. The permutated CGG repeat sequence is prone to expand for offspring (Nolin et al., 1996), and premutation carriers themselves are susceptible to later in life develop conditions considered to be separate from FXS such as fragile X associated tremor and ataxia syndrome (FXTAS, Hagerman et al., 2003) and premature ovarian insufficiency (POI, Schwartz et al., 1994). FMRP is an RNA binding protein (Siomi et al., 1993) that is mainly expressed in the central nervous system (CNS) and gonads, and to lesser extent in other tissues (Hinds et al., 1993). In the CNS, it is expressed in neurons and during development in glial cells. Its expression in astrocytes shows highest levels at the time of vital growth, stabilization, and maturation of synapses, declining thereafter (Pacey and Doering, 2007). FMRP acts as an overall suppressor of protein synthesis (Laggerbauer et al., 2001), a function which it achieves by regulating nuclear-cytoplasmic translocation (Feng et al., 1997; Kim et al., 2009) and translation of various mRNAs. Many of these RNAs are associated with synapse function (Darnell et al., 2011). Hallmarks of neural circuit function in FXS include hyperexcitability and plasticity defects (Martin and Huntsman, 2012). The mouse model of FXS, the *Fmr1* knockout (KO) mice, lacks FMRP and recapitulates the main phenotype, including deficiency in learning and memory, social behavior, and sensory processing (Bassell and Warren, 2008; Liu and Smith, 2009).

Despite several promising therapeutic targets and success with animal models, current therapeutic strategies of FXS remain symptomatic and no specific treatment has reached clinical use. Therapeutic approaches have been based on disturbed mechanisms observed to affect neuron function and the role of non-neuronal cells in these processes has been ignored. The theory around the metabotropic glutamate receptor 5 (mGluR5) proposed that inhibition of the glutamate receptor would rescue downstream ERK1/2 dependent excessive protein synthesis in FXS (Bear et al., 2004); however, treatment resistance likely occurs downstream of targeted factors (Stoppel et al., 2021). The hypercholesterolemia drug 3-hydroxy-3-methylglutaryl coenzyme A (HMG-CoA) reductase inhibitor lovastatin, which inhibits the mevalonate pathway and Ras activation upstream of ERK1/2, was beneficial in preclinical FXS models (Osterweil et al., 2013; Asiminas et al., 2019; Muscas et al., 2019), but did not succeed well in clinical trials (Çaku et al., 2014; Thurman et al., 2020; Champigny et al., 2022). Metformin, targeting ERK1/2 downstream of IGF-1, likewise appeared beneficial in an open-label study and increased GABAergic inhibition (Dy et al., 2018; Proteau-Lemieux et al., 2021). GABA mimetics were studied to influence changes in excitatory/inhibitory balance at circuit level in FXS, and several molecules appeared promising, but the matter of treatment window remains to be resolved (Milla et al., 2023). Selective serotonin uptake inhibitors (SSRIs) and memantine have been found to display beneficial effects (Winarni et al., 2012) and ZYN002 cannabidiol reduced symptoms in a randomized clinical trial (Berry-Kravis et al., 2022). Many therapeutic molecules have been examined on the rescue effects in FXS on dendritic spine abnormalities, which include an increased number of immature spines during early development as a consistent observation (He and Portera-Cailliau, 2013). However, it is unclear to what extent changes in dendritic spine morphology influences functional outcomes in FXS (Booker et al., 2019). Age-, region-, and cell type-specific alterations of structural dynamics of cortical spines are found to be associated with abnormal synaptic plasticity and behavioral deficit in the *Fmr1* KO mice (Cruz-Martín et al., 2010; Pan et al., 2010; Padmashri et al., 2013; Hodges et al., 2017; Gredell et al., 2023). Minocycline was studied on the premise of inhibiting the overactive matrix metalloproteinase 9 (MMP-9) and correcting spine abnormalities in preclinical models with, however, little treatment effect in patients (Utari et al., 2010; Champigny et al., 2022). In general, development of successful treatment strategies in FXS is complicated by the disparity between critical periods of brain plasticity and the age of diagnosis, which is around 4 years for males and 9 years for females (Gabis et al., 2017). Many of the aforementioned treatment approaches for FXS are likely complicated by glial function, and future research on astrocytes has potential to benefit the development of therapies. Here we review astrocytic perspective in FXS.

## 2 Differentiation of FXS astrocytes

Loss of FMRP-mediated translational control results in altered cell fate specification during early neural development (Tervonen et al., 2009; Saffary and Xie, 2011; Utami et al., 2020). Delayed cell cycle progression and extended maintenance of immature proliferating neural progenitor cells (NPCs) are found in both human and mouse FXS models (Edens et al., 2019; Raj et al., 2021). Studying human *FMR1* KO differentiating astrocyte cell cycle dynamics, Ren et al. (2023) observed 2-fold increased cyclin D1 associating with reduced S phase duration in an otherwise unchanged cell cycle duration. The proteomics analysis revealed immature signals in differentiating human iPSC-derived *FMR1* KO astrocytes when compared with controls (Ren et al., 2023). In a separate human *FMR1* KO iPSC cortical model, KO NPCs produced larger organoids and displayed increased glial fibrillary acidic protein (GFAP) expression when compared with isogenic controls (Brighi et al., 2021). The protein kinase inhibitor LX7101 likely through AKT/the mammalian target of rapamycin (mTOR) inhibition was shown to suppress GFAP overexpression (Sunamura et al., 2018). Phospho-SMAD positive astrocytes are increased in *Fmr1* KO cortex at P7, indicating increased bone morphogenetic protein (BMP) activity. Both *Bmp6* RNA and protein are upregulated in rat *Fmr1* KO astrocytes compared with wild type (WT) controls (Caldwell et al., 2022), which can promote astrocyte maturation and inhibit astrocyte proliferation (Scholze et al., 2014). Absence of FMRP appears to influence the dynamics of astrocyte maturation, and some of the mechanisms may be shared with other neurodevelopmental disorders (Caldwell et al., 2022).

In addition to its role in modulating haemostasis and thrombosis, the plasminogen system is implicated in translocation of cellular processes in the developing brain likely through the regulation of the proteolysis of the extracellular matrix (Goeke et al., 2022). Astrocytes regulate plasminogen activation and plasmin clearance in the brain (Briens et al., 2017). Cellular uptake and release processes control plasminogen activators (Cassé et al., 2012) and plasmin substrates (Bergami et al., 2008) in local neuronal environment. Expression of tissue plasminogen activator (tPA) is increased in differentiating *Fmr1* KO neural progenitors, indicating increased tPA activity (Achuta et al., 2014). Colocalising with GFAP, tPA is increased in cortical supragranular layers I-III but decreased in layers IV-V at P7 in the *Fmr1* KO brain (Achuta et al., 2014). Expression of the other main plasminogen activator urokinase-type plasminogen activator (uPA) and its receptor uPAR peak postnatally while being undetectable in the mature brain (Kalderon et al., 1990). uPA is increased in FXS hiPSC-derived forebrain astrocytes (Peteri et al., 2021). These immature human FXS astrocytes also secrete uPA more than healthy control astrocytes, and the increased uPA augments tyrosine receptor kinase B (TrkB) phosphorylation within the docking site for phospholipase-Cγ1 (PLCγ1) in cocultured rat primary neurons (Peteri et al., 2021). Increased production of diacylglycerol (DAG) by PLCγ1 might contribute to increased DAG levels observed in *Fmr1* KO mouse neurons (Tabet et al., 2016) together with reduced diacylglycerol kinase kappa (DGKκ) activity, whose recovery restores behavior in *Fmr1* KO mouse (Habbas et al., 2022). Since uPAR lacks transmembrane/cytoplasmic domains, it needs a partner protein to elicit intracellular signaling. Orphan receptor GPR124 (Chen et al., 2019) is differently expressed in FXS and control astrocytes and may represent a partner receptor that acts through WNT/β-catenin signaling, which is altered in FXS models (Peteri et al., 2021). Astrocytes mature in parallel with other CNS cell types (Cheng Y. T. et al., 2023) and studies of human FXS astrocytes have demonstrated an important impact of the plasminogen system on the differentiation and cell-to-cell interactions of CNS cell types.

## 3 Involvement of astrocytes in FMRP-deficient synapses

### 3.1 Development of synapses

FMRP expression in astrocytes appears essential for normal synapse formation and function (Cheng et al., 2012; Bagni and Zukin, 2019). Co-culturing studies of *Fmr1* KO astrocytes with hippocampal neurons have revealed that FMRP-deficient astrocytes contribute to more complex dendritic arborisation when compared to co-culture with WT astrocytes (Jacobs and Doering, 2010). Dendritic spine immaturity (relatively increased length) and synaptic protein abnormalities of *Fmr1* KO neurons are prevented by WT astrocyte conditioned media or a WT feeder layer (Cheng et al., 2016). Many astrocyte-secreted factors involved in the regulation of synapse formation and maturation are found to be dysregulated in *Fmr1* KO mice. The studies of *Fmr1* KO astrocytes suggest that FMRP deficiency particularly contributes to an abnormal temporal increase in excitatory synapses by modulating secretion of factors that regulate synapse formation and maturation. Tenascin C (TNC), an activator of TLR4 that can promote excitatory synapse formation and immature spine morphology, is overexpressed in *Fmr1* KO astrocytes (Krasovska and Doering, 2018). Also, cortical expression of hevin that is necessary for the formation of thalamocortical excitatory synapses (Kucukdereli et al., 2011; Risher et al., 2014) is increased in P14 *Fmr1* KO mice compared with WT controls consistent with an increase in the density of thalamocortical synapses when WT neurons are cultured with *Fmr1* KO astrocytes (Wallingford et al., 2017). An increased number of thalamocortical synapses occur in layer IV of the somatosensory cortex of 4-month-old *Fmr1* KO mice and altered thalamocortical connectivity is also implicated in ASD (Mizuno et al., 2006; Cheon et al., 2011; Nair et al., 2013). Conversely, expression of SPARC that inhibits the synaptogenic function of hevin and negatively regulates the formation of excitatory synapses (Risher et al., 2014) is reduced in the cortex and hippocampus of the *Fmr1* KO mouse (Wallingford et al., 2017). Secretion of thrombospondin-1 (TSP-1) that as an extracellular matrix protein may induce cell adherence and induce synaptogenesis is also abnormally reduced from *Fmr1* KO astrocytes (Cheng et al., 2016), whereas neurotrophin-3 (NT-3) levels are increased in the secretome of *Fmr1* KO astrocytes as well as in the *Fmr1* KO prefrontal cortex when compared with controls (Yang et al., 2012). Thus, differential spatial and temporal expression pattern of factors secreted from FMRP-deficient astrocytes may reflect astrocyte-dependent brain region-specific developmental processes. Dysregulation of secreted factors from FMRP-deficient astrocytes during critical periods for synapse formation, stabilization, and pruning are consistent with delayed critical period for thalamocortical plasticity in the barrel cortex (somatosensory layer IV) of *Fmr1* KO mice and with increased silent synapses at earlier time points (Harlow et al., 2010).

### 3.2 FMRP-deficient astrocytes and synapse function

Subcellular localization/expression of mRNAs and localization of ribosomes at perisynaptic astroglial processes (PAPs) are critical for localized astroglial signaling to synapses and regulation of neuronal activity (Bazargani and Attwell, 2016). There is evidence that astroglial processes have more contacts with synapses in the cortex of the *Fmr1* KO mice than in WT controls (Men et al., 2022). FMRP deficiency affects localization of mRNAs in astrocytes and particularly alters levels of mRNAs localized at astrocytic processes. The mRNAs located at processes are enriched by mRNAs that encode surface proteins such as transporters, receptors, and channels involved in synaptic signaling (Men et al., 2022). Effects of the absence of FMRP on the total mRNA or on the local mRNA amount may serve as a critical factor for activity-dependent regulation of synapses.

Increased glutamatergic signaling and altered excitatory/inhibitory balance is recognized as a hallmark of FXS (Liu et al., 2022). The role of FXS astrocytes in abnormal neuronal firing was recently confirmed in co-cultures of cortical neurons and astrocytes derived from human stem cells generated from patients with FXS and a control donor (Das Sharma et al., 2023). FXS neurons showed a high frequency of short, spontaneous bursts and reduced persistent Na^+^ current in the presence of FXS astrocytes or media secreted by FXS astrocytes. Astrocyte-specific Ca^2+^ binding protein S100B was found to prevent abnormalities of Na^+^ current and firing pattern in FXS and control neurons co-cultured with FXS astrocytes or FXS astrocyte-derived conditioned medium. Furthermore, blocking antibody to S100B induced disordered firing in co-cultures of control neurons and control astrocytes (Das Sharma et al., 2023), demonstrating that astrocyte secreted factors can influence the firing patterns of glutamatergic neurons (Das Sharma et al., 2020).

An important role of astrocytes is to maintain glutamate homeostasis that is essential for brain physiology. Astrocyte-specific *Fmr1* KO mouse displays reduced expression of the astrocytic glutamate transporter GLT-1, which leads to reduced astrocytic uptake of extracellular glutamate and increased cortical neuronal excitability (Higashimori et al., 2016). Astroglial glutamate transporters modulate activation of neuronal mGluR1/5 providing a mechanism, which may underlie enhanced neuronal mGluR5 activation in FXS. mGluR5 regulates GLT-1 expression in astrocytes and reduced mGluR5 expression has been found both in human and mouse FMRP-deficient astrocytes (Higashimori et al., 2013; Men et al., 2020). Selective astroglial overexpression of miRNA-128 results in reduced mGluR5 protein in FMRP-deficient astrocytes (Men et al., 2020). Regulation of astroglial transporter expression is strongly associated with astrocytic maturation and influenced by culturing conditions (Schlag et al., 1998; Peteri et al., 2021), which may explain that Ren and colleagues (Ren et al., 2023) did not observe reduced glutamate uptake in human *FMR1* KO astrocytes. Species specific differences and differences in experimental approaches such as glutamate incubation time may explain different results in studies on astrocytic glutamate uptake (Higashimori et al., 2016; Ren et al., 2023). Astrocytic expression of mGluR5 is developmentally regulated and possibly several compensatory mechanisms also exist as mGluR5 inhibition can lead to increased GLT-1 expression under pathological conditions (Cheng S. et al., 2023). To what extent compensatory mechanisms in glutamate uptake by FXS astrocytes are dependent on cell-to-cell interactions and neuronal activity-dependent mechanisms remain to be studied.

In addition to the glutamatergic system, GABAergic circuits and GABA homeostasis are dysregulated in the absence of FMRP. Reduction of several GABA receptor subunits in various brain regions associates with reduced expression of GABA metabolizing enzymes and reduced numbers of parvalbumin (PV) positive neurons in the *Fmr1* KO mouse (Gao et al., 2018; Van der Aa and Kooy, 2020). Contribution of FMRP-deficient astrocytes to the aberrant GABA phenotype was indicated by an astrocyte-specific postnatal *Fmr1* KO model (Rais et al., 2022, preprint available). Loss of astrocytic FMRP recapitulated the reduced number of cortical PV cells and reduced synaptic GABA_*A*_ receptor subunits (Rais et al., 2022, preprint available). These changes associated with increased GABA levels and increased glutamate decarboxylase in astrocytes, indicating increased GABA synthesis (Rais et al., 2022, preprint available). Astrocytes can sense extracellular GABA as well as release GABA through ion channels and transporters, controlling its extracellular levels (Kilb and Kirischuk, 2022), suggesting that increased astrocytic GABA in *Fmr1* KO mice may be physiologically compensatory to maintain homeostasis.

Astrocytes exhibit intracellular Ca^2+^ currents which can modulate synapse function by altering gliotransmitter release (Goenaga et al., 2023). There is ample evidence that these Ca^2+^ signals are altered in the context of FMRP-deficient astrocytes. Ca^2+^ signals in response to extracellular adenosine triphosphate (ATP) in human *FMR1* KO astrocytes have increased amplitude but neither the proportion of cells responding nor the duration of the response change (Ren et al., 2023). Augmented intracellular Ca^2+^ responses to membrane depolarization with high extracellular K^+^ are dependent on tPA in *Fmr1* KO mouse NPCs (Achuta et al., 2014). In human FXS iPSC-derived astrocytes, L-type calcium channel-dependent Ca^2+^ responses to high extracellular K^+^ were reduced when compared with controls and these responses showed strong inverse correlation with ACM uPA levels (Peteri et al., 2021), demonstrating that altered Ca^2+^ signaling in FXS astrocytes modulates neuronal plasticity.

## 4 GFAP and astrocyte reactivity in FXS

Astrocytes with abnormally high GFAP, have been observed in striatal, hippocampal, cortical, and cerebellar areas of adult *Fmr1* KO mice (Yuskaitis et al., 2010; Pacey et al., 2015). Increased GFAP expression is also found in a human *FMR1* KO iPSC cortical organoid model (Brighi et al., 2021). In human FXS cortical layer I GFAP and S100B positive cells are increased (Ren et al., 2023). Whether increased GFAP expression in FXS astrocytes reflects altered astrocyte fate determination, astrocyte immaturity or response of astrocytes to extracellular stressors remains to be elucidated.

Astrocytes with abnormally high GFAP reflecting astrocyte reactivity occurs in response to various genetic, environmental, and pathological stimuli (Escartin et al., 2021). Pacey et al. (2015) showed that astrocytes in the cerebellum of *Fmr1* KO mice display chronic, persistent activation with little or no activation of microglia. Expression of glial cell markers tumor necrosis factor receptor 2 (TNFR2), leukemia inhibitory factor (LIF), and GFAP were found to be elevated in the *Fmr1* KO mouse cerebellum at 2 weeks postnatally. GFAP, TNFR2, and S100B were increased in the adult *Fmr1* KO brain (Pacey et al., 2015). The proinflammatory cytokine interleukin 1β (IL-1β) is an activator of human astrocytes leading to production of inflammatory mediators such as cytokines, chemokines, nitric oxide (NO), and reactive oxygen species (ROS) (Lee et al., 1993; Sharma et al., 2007) and to down-regulation of glutamate uptake (Hu et al., 2000). IL-1β treatment increases *GFAP* and chemokine *CCL5* mRNA expression more in human FXS iPSC-derived astrocytes than in control astrocytes (Talvio et al., 2023), suggesting that FXS astrocytes are characterized by increased sensitivity to reactivity. This may be reflected in *Fmr1* KO mice as impaired brain energy metabolism and increased ROS markers (el Bekay et al., 2007; D’Antoni et al., 2020). Mitochondrial respiration capacity and emission of reactive oxygen species are increased in *Fmr1* KO mouse astrocytes under physiological hypoxia but not in atmospheric normoxia (Vandenberg et al., 2022). In the transcriptomics analysis of human FXS NPCs, MYD88 was amongst the most significantly overexpressed genes (Talvio et al., 2022) and there are several tentative arguments for MYD88-mediated system overactivity in FXS astrocytes. Expression of MMP-9, the proposed target of minocycline in FXS, is upregulated via a MYD88-dependent mechanism in astrocytes (Gorina et al., 2011), and reduced IL-10 in FXS astrocytes (Talvio et al., 2023) might contribute to increased MYD88 (Chang et al., 2009). TLR4/MyD88-mediated induction of IL-6 is overactive in *Fmr1* KO astrocytes (Krasovska and Doering, 2018), implicating involvement of inflammatory factors and suggesting alteration in TLR4/MyD88/PI3K interactions (Laird et al., 2009) in FXS astrocytes.

## 5 Altered lipidome of FXS astrocytes

Cholesterol is an essential component of cell membranes, where it influences membrane fluidity and the formation of signaling microdomains (signaling rafts), such as what occur in dendritic spines (Hering et al., 2003). Cholesterol is also the precursor of neurosteroids (Lloyd-Evans and Waller-Evans, 2020), and it participates in signaling on its own by binding membrane proteins (Sheng et al., 2012). Cholesterol does not pass the blood brain barrier, so the CNS pool is maintained locally. Neurons and astrocytes synthesize cholesterol in development, but as neurons acquire maturity and brain total cholesterol reaches a plateau stage, astrocytes become largely responsible for maintaining lipid homeostasis in the brain (Jin et al., 2019; Chen et al., 2023). There is evidence that astrocytic cholesterol stimulates the formation of synapses in radial glial cell cultures (Mauch et al., 2001).

A recently published extensive work by Ren and colleagues (Ren et al., 2023) on human stem cell-derived astrocytes identified cholesterol synthesis pathway to be downregulated in the proteomics analysis. Increased lanosterol synthase and lanosterol, an intermediate oxysteroid in the Bloch pathway of cholesterol synthesis, were associated with reduced downstream enzymes and total cholesterol in three out of the four FXS cell lines studied and compared with their isogenic controls. In studies of *Fmr1* KO mouse astrocytes, cholesterol, and desmosterol (the last precursor of cholesterol in the Bloch pathway) accumulated in astrocytes (Talvio et al., 2023). Here, cholesterol levels did not differ between human or mouse FMRP-deficient and control astrocyte conditioned medium, suggesting that astrocytes maintained extracellular cholesterol homeostasis to avoid cholesterol toxicity (Adachi et al., 2022; Talvio et al., 2023). Both mouse and human FMRP-deficient astrocytes expressed less ATP-binding cassette transporter A1 (ABCA1, the main astrocytic cholesterol exporter) than their respective controls. Reduced ABCA1 expression associated with reduced secretion of IL-13 and IL-10, which are implicated in ABCA1 regulation (Cardilo-Reis et al., 2012; Ma et al., 2012). Since astrocyte culturing conditions and differentiation protocols for production of iPSC-derived astrocytes can affect lipidomics of astrocytes, they may explain differences in the cholesterol balance observed in different FXS astrocyte models (Ren et al., 2023; Talvio et al., 2023). Leukemia inhibitory factor LIF is a factor used to specify astrocytes during differentiation (Ren et al., 2023) and by inducing ABCA1 expression it might modulate cholesterol secretion (Trouillas et al., 2009).

There are several changes in the lipidome of the *Fmr1* KO mouse astrocytes (Talvio et al., 2023). ABCA1 affects membrane structure by transporting cholesterol, sphingomyelin, phosphatidylserine, and phosphatidylcholine (PC) species from the cytoplasmic to the exocytoplasmic leaflet (Quazi and Molday, 2013). PC species, which are the most common membrane phospholipids, were the most differently regulated membrane lipid class in the *Fmr1* KO astrocyte lipidomics analysis (Talvio et al., 2023). *Fmr1* KO astrocytes contained more of the most unsaturated PC species (Talvio et al., 2023). Mutual aversion by cholesterol and polyunsaturated fatty acids (PUFA) drives the segregation of membrane microdomains (Wassall and Stillwell, 2009). The capacity of the *Fmr1* KO astrocyte membranes to buffer cholesterol overload and prevent cholesterol toxicity was compromised due to the phospholipid profile containing highly unsaturated PC species and less sphingomyelin (Talvio et al., 2023). Further studies are needed to determine FXS-specific lipid changes in astrocytes more broadly.

## 6 Discussion

**FIGURE 1:**
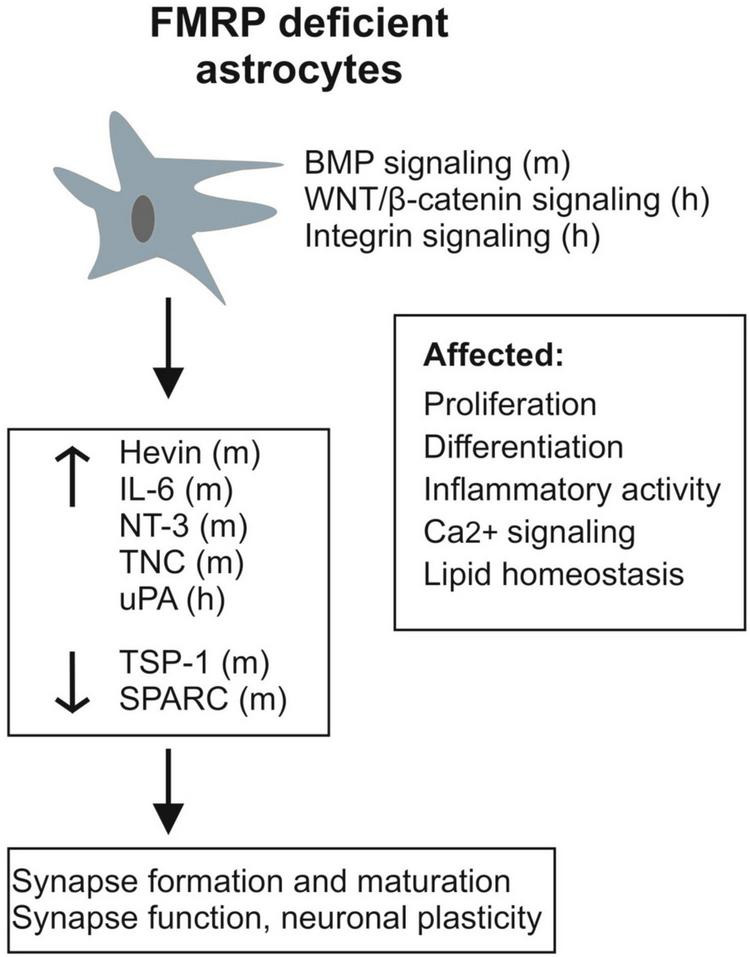
Schematic presentation summarizing alterations observed in FMRP-deficient astrocytes and their secretome (h, human; m, mouse). In addition to changes in specific known astrocytic molecules influencing synapses, various signaling pathways (BMP, WNT/β-catenin, and integrin signaling) involved in brain development and many aspects of brain homeostasis are differently regulated in FXS astrocytes.

Astrocytes express FMRP during development (Pacey and Doering, 2007; Gholizadeh et al., 2015), and its absence influences the way astrocytes differentiate in both human and murine models of FXS. As summarized in Figure 1, studies of the mouse *Fmr1* KO astrocytes and human stem cell-derived astrocytes have shown a number of differences between FMRP-deficient and healthy control astrocytes, including changes in factors secreted from astrocytes as well as transcriptomics and protein expression profiles, lipidomics, Ca^2+^ signaling, and inflammatory activity of astrocytes. Both human and mouse FMRP-deficient astrocytes display modulation of the plasminogen system, which can influence the development of CNS cell types. Astrocytes sense and respond to changes in the extracellular milieu, and studies on the *Fmr1* KO mouse astrocytes have revealed alterations in astrocyte released factors that differentially regulate excitatory synapse development and how astrocytes influence glutamatergic signaling in the absence of FMRP. Altered cholesterol balance in *Fmr1* KO mouse astrocytes together with increased levels of PUFA may contribute to the capacity of astrocytes to maintain extracellular homeostasis by modulating membrane properties and influencing receptor functions.

The *Fmr1* KO mouse model of FXS parallels the phenotype of FXS, and studies on the mouse model have shed light on the role of FMRP in CNS development and function. However, for instance, cognitive impairment and anxiety of the FXS phenotype are not fully recapitulated in the mouse model (Kazdoba et al., 2014). Human astrocytes are more complex than their mouse counterparts, and many abnormalities observed in *Fmr1* KO astrocytes need to be replicated in human models. Patient-derived iPSC and *FMR1* KO human stem cells provide a tool to model human astrocytes. However, mature astrocytes represent heterogeneous populations (Oberheim et al., 2012) and they mature in conjunction with associated neurons (Cheng Y. T. et al., 2023), indicating that neuronal contacts and neuronal differentiation are necessary for astrocyte maturation. In the absence of full understanding of astrocytic specialization and the processes that govern it, astrocyte monocultures are likely to exhibit immature astrocytes and simple co-culture setups fail to model the range of astrocytes needed in order to form global conclusion about astrocytic dysfunction. *In vitro* human organoid models may overcome the issue of modeling astrocytic variability and the *Fmr1* KO murine models facilitate replication of results *in vivo*.

## Author contributions

KT: Writing – review & editing. MC: Writing – review & editing.
